# Supplementary material for: Phytochemical Analysis, In Vitro Anticholinesterase, Antioxidant Activity and In Vivo Nootropic Effect of Ferula ammoniacum (Dorema ammoniacum) D. Don. in Scopolamine-Induced Memory Impairment in Mice
Source: Brain Sci. 2021 Feb 19;11(2):259. doi: 10.3390/brainsci11020259 (PMC7922987; doi:10.3390/brainsci11020259)
Supplement: Supplementary file 1 [file brainsci-11-00259-s001.pdf]

Supplementary Material

Table S1. Percent AChE and BChE inhibition potential of *Ferula ammoniacum* (D. Don) seed extracts at various concentrations.

S. No	Sample	Concentration (µg/mL)	% AChE Mean ± SEM	AChE IC_{50} (µg/mL)	% BChE Mean ± SEM	BChE IC_{50} (µg/mL)
1	Fa.Met	1000	66.51 ± 0.71***	80	62.18 ± 0.96***	95
		500	60.32 ± 0.83***		59.39 ± 1.19***	
		250	55.48 ± 1.18***		54.53 ± 1.15***	
		125	50.45 ± 1.75***		51.28 ± 0.40***	
		62.5	47.32 ± 0.85**		48.56 ± 1.16***	
		31.05	45.67 ± 0.95*		34.79 ± 0.60**	
2	Fa.Hex	1000	45.41 ± 1.15***	215	50.08 ± 0.87***	205
		500	41.51 ± 1.09***		45.64 ± 0.91***	
		250	38.63 ± 0.84***		40.22 ± 1.55***	
		125	34.66 ± 0.69***		37.26 ± 0.57***	
		62.5	32.11 ± 1.16***		32.52 ± 0.51***	
		31.05	30.39 ± 1.42**		29.33 ± 1.15***	
3	Fa.Chf	1000	88.44 ± 0.80*	43	85.31 ± 0.49**	42
		500	84.93 ± 2.19**		78.67 ± 1.26**	
		250	78.94 ± 1.45**		75.96 ± 0.73*	
		125	67.36 ± 1.58**		68.55 ± 1.12 *	
		62.5	58.84 ± 1.07**		55.28 ± 0.77 *	
		31.05	43.08 ± 1.29*		46.51 ± 0.59 ^{ns}	
4	Fa.EtAc	1000	89.22 ± 1.74*	40	86.37 ± 0.61 **	41
		500	75.58 ± 1.54**		78.65 ± 0.77 **	
		250	72.18 ± 1.08**		74.71 ± 0.97 **	
		125	68.95 ± 0.86**		62.64 ± 1.17 **	
		62.5	52.14 ± 1.04**		58.44 ± 1.12*	
		31.05	46.72 ± 1.15 ^{ns}		48.91 ± 0.98 ^{ns}	
5	Fa.Bn	1000	35.03 ± 0.78***	330	40.11 ± 0.67***	325
		500	30.66 ± 0.72***		36.27 ± 0.49***	
		250	29.28 ± 0.77***		30.11 ± 1.15***	
		125	21.33 ± 1.85***		28.31 ± 1.22***	
		62.5	19.46 ± 1.09***		21.27 ± 0.64***	
		31.05	11.29 ± 0.99***		18.74 ± 1.15***	
6	Fa.Aq	1000	50.66 ± 1.50***	200	55.66 ± 0.69 ***	195
		500	45.08 ± 1.32***		50.36 ± 0.38 ***	
		250	40.54 ± 1.10***		47.80 ± 0.81 ^{ns}	
		125	38.48 ± 0.45***		42.59 ± 0.71 *	
		62.5	36.01 ± 1.15***		39.64 ± 0.84 ***	
		31.05	30.31 ± 1.01***		36.33 ± 0.57 ^{ns}	
7	Standard Galantamine	1000	96.56 ± 1.08	30	95.17 ± 0.71	32
		500	91.90 ± 1.36		88.44 ± 0.62	
		250	87.78 ± 1.22		80.73 ± 0.95	
		125	75.11 ± 0.62		71.64 ± 0.23	
		62.5	67.59 ± 1.36		66.38 ± 1.14	
		31.05	49.11 ± 0.58		50.52 ± 0.88	

Abbreviations: Fa, *Ferula ammoniacum;* AChE, Acetylcholine esterase; BChE, Butyrylcholine esterase; Fa. Met, Crude methanolic extract; Fa. Hex, *n*-hexane fraction; Fa. Chf, Chloroform fraction; Fa.EtAc, Ethyl acetate fraction; Fa. Bn, *n*-Butanol; Fa. Aq, Aqueous fraction

Note: The data is represented as mean ± SEM, (N = 3). Values are significantly different as compare to positive control Galantamine, *P < 0.05, **P < 0.01, ***P < 0.001.

Table S2. Percent DPPH and ABTS free radical scavenging activity of *Ferula ammoniacum* (D. Don) seed extracts at various concentrations.

S. No	Sample	Concentration (µg/mL)	% DPPH Scavenging	IC_{50} (µg/mL)	% ABTS Scavenging	IC_{50} (µg/mL)
			Mean ± SEM		Mean ± SEM	
1	Fa.Met	1000	71.91 ± 0.55***		58.48 ± 0.96***	
		500	62.01 ± 0.56***		50.26 ± 0.62***	
		250	42.49 ± 0.44***	140	32.71 ± 0.87***	250
		125	40.75 ± 0.88***		27.44 ± 0.85***	
		62.5	36.22 ± 0.61***		16.55 ± 0.81***	
		31.05	32.47 ± 0.65**		14.30 ± 0.35***	
		1000	34.58 ± 0.90***		36.04 ± 0.57***	
2	Fa.Hex	500	30.22 ± 0.61***		34.32 ± 0.87***	
		250	28.09 ± 0.59***	920	31.10 ± 0.53***	910
		125	24.64 ± 0.86***		29.35 ± 0.32***	
		62.5	19.97 ± 0.55***		23.95 ± 1.02***	
		31.05	15.84 ± 0.89***		17.11 ± 0.49***	
3	Fa.Chf	1000	73.23 ± 1.55***		66.19 ± 0.60***	
		500	66.88 ± 0.59***		60.35 ± 0.63***	
		250	60.81 ± 0.69***	120	54.22 ± 0.59**	210
		125	52.98 ± 1.14***		50.72 ± 0.64**	
		62.5	49.89 ± 0.45***		47.12 ± 0.47*	
		31.05	45.25 ± 0.60***		43.71 ± 1.18^{n}	
		1000	81.86 ± 0.59**		75.08±0.57***	
4	Fa.EtAc	500	73.49 ± 0.84**		62.29±0.66***	
		250	62.30 ± 1.30***	100	59.31±0.74**	120
		125	52.60 ± 0.93**		54.45±0.88**	
		62.5	44.48 ± 1.27**		50.04±1.51*	
		31.05	32.27 ± 1.08*		45.38±0.86*	
		1000	36.57 ± 0.91***		44.73 ± 0.79***	
5	Fa.Bn	500	33.65 ± 0.71***		40.57 ± 0.55***	
		250	29.29 ± 0.64***	910	36.09 ± 0.58***	870
		125	23.62 ± 1.18***		31.22 ± 1.17***	
		62.5	20.55 ± 0.66***		27.81 ± 0.83***	
		31.05	18.07 ± 1.48 *		21.54 ± 1.08**	
6	Fa.Aq	1000	39.83 ± 1.15***		47.73 ± 0.91***	
		500	35.46 ± 0.86***		44.09 ± 0.88***	
		250	31.91 ± 1.06***	900	30.68 ± 0.80***	860
		125	28.51 ± 0.84***		38.62 ± 0.74***	
		62.5	24.78 ± 0.66***		32.07 ± 0.49***	
		31.05	20.76 ± 0.62**		29.82 ± 1.17**	
		1000	91.32 ± 0.34		95.31 ± 0.75	
7	Ascorbic	500	87.26 ± 0.59		82.79 ± 1.10	
	acid	250	80.19 ± 1.15	30	77.33 ± 0.67	45
		125	67.98 ± 1.14		62.92 ± 0.81	
		62.5	54.90 ± 1.01		53.55 ± 1.01	
		31.05	27.54 ± 0.84		43.64 ± 0.69	

Abbreviations: Fa, *Ferula ammoniacum;* Fa.Met, Crude methanolic extract; Fa. Hex, *n*-hexane fraction; Fa.Cf, Chloroform fraction; Fa.EtAc, Ethyl acetate fraction; Fa.Bn, *n*-Butanol; Fa. Aq, Aqueous fraction.
The data is represented as mean ± SEM, (N = 3). Values are significantly different as compare to positive control Ascorbic acid, *P < 0.05, **P < 0.01, ***P < 0.001.

Table S3. Nootropic effect of *Ferula ammoniacum* (D. Don) seeds extracts in Novel Object Recognition Test.

**Treatments**	**Exploration time (seconds)**			**Discrimination index**	**Percent Discrimination index**
	**Session 1**	**Session 2**			
		**N**	**F**		
DW+Sal[a]	39.2	36.0±2.1	11.00±1.1	0.53	53
DW+Scop[b]	20.4	25.0±1.6***	18.00±2.4***	0.16***	16
Fa.EtAc 50+Scop[c]	26.9	36.5±2.2#	15.10±2.5##	0.41##	41
Fa.EtAc 100+Scop[c]	27.5	37.3±3.5#	14.20±3.1#	0.45##	45
Fa.EtAc 200+Scop[c]	31.5	38.5±2.2#	12.40±2.3#	0.51#	51
DZP+Scop[c]	34.2	34.2±1.2##	11.30±1.5#	0.50#	50

Abbreviations: Fa, *Ferula ammoniacum;* Fa.EtAc, Ethyl acetate fraction. [The data is expressed as Mean ± SEM, Each value corresponds to a mean of eight animals. One way ANOVA followed by Dunnett's *post hoc* multiple comparison test to determine the values of P. ***p < 0.001; comparison of DW+Sal[a] (Normal control) vs DW+Scop[b] (Scopolamine treated group), #p < 0.05 and ##p < 0.01; comparison of (DW+Scop)[b] vs DZP+Scop[c] (Donepezil treated) and Fa.EtAc[c] (50, 100 and 200 mg/kg) treated groups using one way ANOVA followed by Dunnett's post hoc multiple comparison test].
